# Resilience of Central Pacific reefs subject to frequent heat stress and human disturbance

**DOI:** 10.1038/s41598-019-40150-3

**Published:** 2019-03-05

**Authors:** Simon D. Donner, Jessica Carilli

**Affiliations:** 1 Department of Geography, 1984 West Mall University of British Columbia, Vancouver, British Columbia V6T 1Z2 Canada; 2 Australian Nuclear Science and Technology Organization, New Illawarra Rd, Lucas Heights, NSW 2234 Australia; 3 Present Address: Energy and Environmental Sciences, Space and Naval Warfare Systems Center Pacific, 53475 Strothe Rd, San Diego, CA 92152 USA

## Abstract

Frequent occurrences of coral bleaching and associated coral mortality over recent decades have raised concerns about the survival of coral reefs in a warming planet. The El Niño-influenced coral reefs in the central Gilbert Islands of the Republic of Kiribati, which experience years with prolonged heat stress more frequently than 99% of the world’s reefs, may serve as a natural model for coral community response to frequent heat stress. Here we use nine years of survey data (2004–2012) and a suite of remote sensing variables from sites along gradients of climate variability and human disturbance in the region to evaluate the drivers of coral community response to, and recovery from, multiple heat stress events. The results indicate that the extent of bleaching was limited during the 2009–2010 El Niño event, in contrast to a similar 2004–2005 event, and was correlated with incoming light and historical temperature variability, rather than heat stress. Spatial and temporal patterns in benthic cover suggest growing resistance to bleaching-level heat stress among coral communities subject to high inter-annual temperature variability and local disturbance, due to the spread of “weedy” and temperature-tolerant species (e.g., *Porites rus*) and the cloudy conditions in the region during El Niño events.

## Introduction

Episodes of heat stress and subsequent mass coral ‘bleaching’ over the past three decades have led to widespread coral mortality and raised questions about the viability of coral reef ecosystems during a period of rapid climate change^1–4^. Elevated sea surface temperatures (SSTs) of only 1–2 °C above the usual local maximum can lead to coral bleaching, visually apparent as a loss of colour from the reef-building animals due to a breakdown of the symbiosis with the colourful dinoflagellate *Symbiodinium* that reside in coral tissue. The response of coral communities to repeated thermal stress will depend on the capacity of individual corals and their symbionts for physiological acclimatization^5^, directional selection to more heat-tolerant genotypes^6^, shifts in the abundance of more heat-tolerant symbionts^7,8^, as well as shifts to more heat-tolerant coral taxa due to selective mortality^9,10^. Locations with natural exposure to unique modes of temperature variability may provide insight into the ability of individual corals and/or coral communities to adjust to a rising frequency and severity of heat stress^11^.

**Figure 1:**
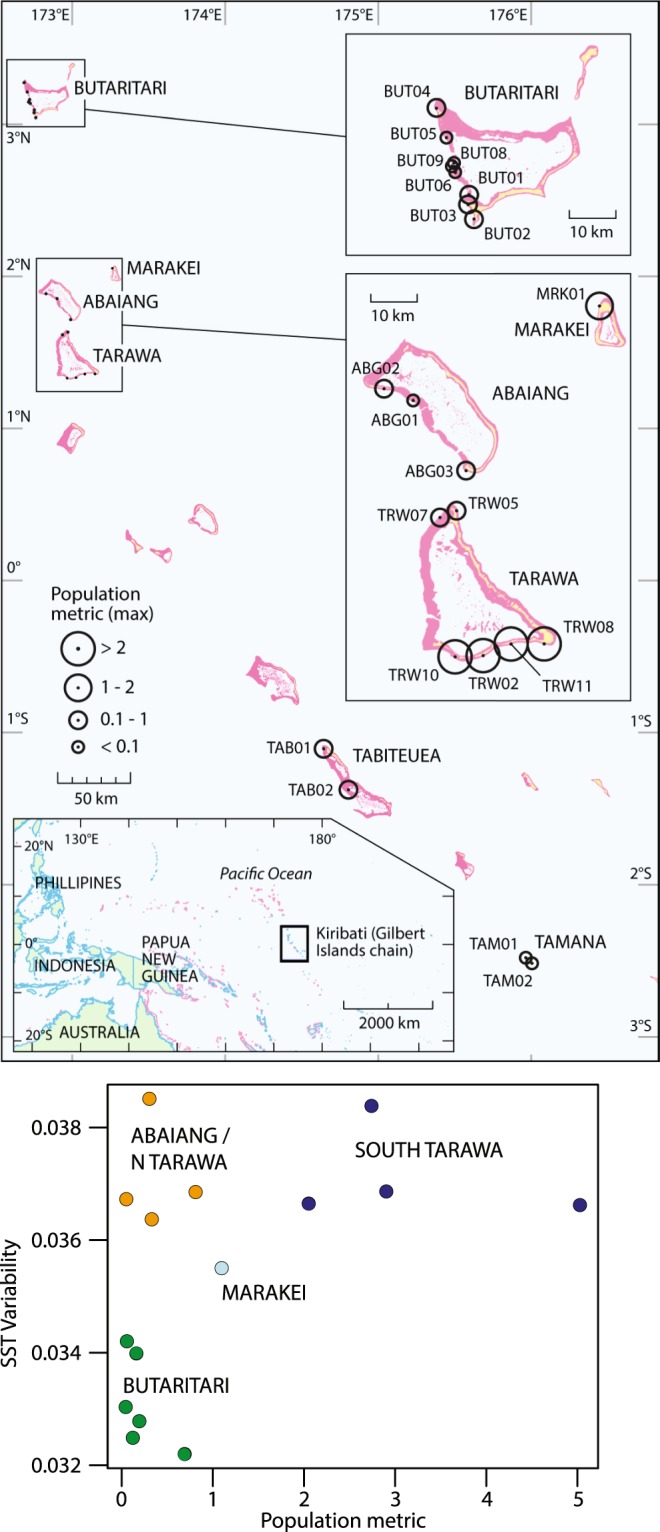
Study region in the Gilbert Islands of the Republic of Kiribati. Top panel (map) shows the field sites and a metric of adjacent human population; bottom plot contrasts coefficient of variability of weekly sea surface temperature (SST) vs. the population metric for the N. Gilbert sites. Kiribati (kee-ree-bas) is the local pronunciation of Gilberts, the colonial name for the main island chain. Gilbert Islands is used here to avoid confusion between the particular island chain and the entire country.

An understudied set of reefs exposed to unique climate variability lies in the Gilbert Islands of the Republic of Kiribati (Fig. 1). Like other equatorial reefs, the Gilbert Islands experience consistently high insolation and temperatures throughout the year; the SST in Tarawa Atoll (~1°N) varies by only roughly 2 °C throughout the year. However, unlike many other equatorial reefs, the coral reefs around Tarawa and its neighbour Abaiang Atoll experience year-to-year variability in climate, including winds, currents, SSTs, cloudiness, and rainfall, due to the El Niño/Southern Oscillation (ENSO). As a consequence, SSTs in the Gilbert Islands vary twice as much from year-to-year than from season-to-season, unlike 99% of the world’s coral reefs^12^. This atypical temperature variability causes bleaching-level heat stress, according to standard metrics like the National Oceanic and Atmospheric Administration (NOAA) Coral Reef Watch’s Degree Heating Weeks (DHW) product, to occur at a frequency^12^ that other coral reefs are unlikely to experience without several decades of further warming^13^. For these reasons, the reefs surrounding the central Gilbert Islands may serve as a model for how coral communities withstand frequent heat stress.

Within the region, latitudinal differences in ENSO influence and human settlement patterns create gradients in year-to-year climate variability and human disturbance^14,15^ (Fig. 1). Relatively urbanized South Tarawa experiences high ENSO-driven SST variability and high human disturbance. It is home to roughly half the country’s population (50,182 people, 3,184/km^2^; 2010 census^16^) and reef sites there are all subject to sewage pollution due to poor sanitation (e.g., 16% of households have flush toilets on the public sewer system^16^), sedimentation from shoreline construction, poor lagoon flushing due to causeway construction, and exploitation for reef rock and food resources^17–19^. Nearby Abaiang (5,502 people; 344/km^2^) experiences similar SST variability to South Tarawa but has a small population that engages largely in subsistence activities, resulting in comparatively lower sewage runoff, sedimentation, and fishing pressure onto the reefs. By contrast, the reefs around more northern Butaritari Atoll (~3°N) experience limited human disturbance (4,346 people; 322/km^2^) but lower SST variability than all other atolls. Despite being only 180 km north of Tarawa, Butaritari lies on the edge of the influence of the South Equatorial Current (SEC) and therefore experiences less of a climate response to ENSO.

The unique geography of the northern Gilbert Islands presents an opportunity to examine the influence of both climate variability and local human disturbance on coral response to ENSO-driven heat stress events. Past benthic monitoring in the Gilbert Islands, however, has been limited due to physical isolation, the available resources for underwater work, the lack of local science capacity, and the rough ocean conditions between atolls and on windward reefs^14^. In a study of cores and tissue samples from massive *Porites sp*. corals, Carilli *et al*.^15^ reported evidence of greater bleaching resistance to 2004–2005 and 2009–2010 heat stress events at high SST variability sites in Tarawa and Abaiang, with a small negative influence associated with local human disturbance, compared to lower SST variability sites in Butaritari. Yet the response of the coral community over time across the different atolls is still not well understood.

**Figure 2:**
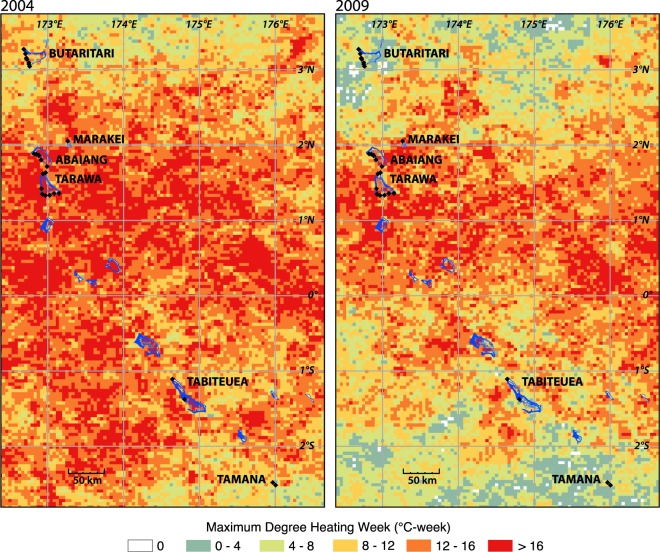
Maximum annual Degree Heating Week (°C·week) value across the Gilbert Islands during the a) 2004–2005 and b) 2009–2010 events. In both cases, the events persisted into the second year, but maximum values occurred in November and December of the first year.

Here we examine benthic data collected periodically from 2004 through 2012 (see Materials and methods section) to evaluate the influence of climate variables and local human disturbance on coral community composition. First, we compare the effect of the 2004–2005 and 2009–2010 heat stress events (Fig. 2) using data from five sites in South Tarawa and Abaiang for which shallow water (3–5 m) data is available back to 2005. Second, we examine the relationship between the observed bleaching during the 2009–2010 heat stress event and a suite of physical and biological variables derived from remote sensing across a more extensive set of sites surveyed in 2010. Third, we evaluate the influence of both climate variability and human disturbance on the trajectory of the coral community using data from the 2010 surveys and more extensive 2012 surveys. Though limited by the opportunistic nature of benthic monitoring in the Gilbert Islands, this analysis reveals resistance to bleaching-level heat stress among highly disturbed coral communities subject to frequent past temperature variability, likely due to the spread of weedy corals (*Porites rus*) and other temperature-tolerant taxa, and the protection afforded by low light conditions during ENSO events.

## Results

### Response to 2004–2005 vs. 2009–2010 heat stress events

According to the available shallow data from the five sites at Tarawa (TRW02, TRW08) and Abaiang (ABG01, ABG02, ABG03), the bleaching response was more limited during the 2009–2010 heat stress event than the 2004–2005 event. The bleaching response index (BRI) across the five sites was significantly higher (t = 5.43, p = 0.005, ΔBRI = 0.38; see Materials and methods section) in the 2005 data than the 2010 data (Table S1). The BRI by taxa was also lower in 2010 for each of the major taxa present at all sites; the difference was greatest for *Pocillopora* (ΔBRI = 0.82) which experienced marked decline in abundance after the 2004–2005 event^14^ (Table S1). The bleaching response was significantly lower during the 2009–2010 event despite similar levels of heat stress to the 2004–2005 event. Although the 2004–2005 event reached higher maximum DHW values (mean of 15.3 °C-week vs. 12.3 °C-week) and lasted longer (22 weeks vs. 17 weeks at Alert Level I) on average across the five sites than the 2009–2010 event (Fig. 2; Table S2), the differences were not statistically significant (maximum DHW, p = 0.32; weeks of Alert Level I, p = 0.20).

Benthic cover data further illustrates the limited effect of the 2009–2010 heat stress event, relative to the 2004–2005 event (Table S3). Live hard coral cover increased on average by 9.3% from 2005 to 2009 across the five sites, followed by a decrease of 3.0% from 2009 to 2010, and a subsequent decrease of 3.1% from 2010 to 2012, however none of these trends were significant. The three sites which exhibited increases in coral cover from 2005 to 2009 (TRW08, ABG01, ABG03) were also the only sites that exhibited decreases over the 2009–2010 heat stress event. The difference in coral community response to the 2004–2005 and 2009–2010 events is further evident in the cover of recently dead and bleached coral. Together, these categories comprised from 25% to 66% of all (live and dead) coral cover at the different sites in 2005 surveys, but only 3% to 7% of all coral in the 2010 surveys. The fraction of recently dead and bleached coral cover in the 2010 surveys was similarly low at 10–12 m depth (6% to 10% of all coral), as well as at a North Tarawa site (TRW07) added that year (5% at 3–5 m, 8% at 10–12 m).

### Drivers of bleaching during the 2009–2010 stress event

The results of the more extensive 2010 surveys, which included Butaritari and the southern Gilbert Islands of Tamana and Tabiteuea, indicate that sites further from the equator experienced greater bleaching despite lower heat stress exposure. The BRI from the 2009–2010 event ranged from low values of 0.01 to 0.08 at Tarawa and Abaiang sites, through moderate values of 0.07 to 0.28 at Butaritari sites, to high values of 0.36 to 0.59 at Tamana and Tabiteuea (Table S4). Among the remote sensing variables, BRI is most significantly positively correlated with photosynthetically active radiation (PAR) during the 2009–2010 boreal winter (Table 1). There were also positive correlations with wave height and maximum monthly mean (MMM), although not significant with the Bonferroni correction. There were strongly negative relationships (p < 0.01) between BRI and several metrics of past SST variability and past heat stress, including the coefficient of variation of weekly SSTs (CV_SST_), the mean number of weeks with DHW >4 °C-week (WkDHW4_avg_), and the fraction of past years with DHW > 4 °C-week (fDHW4); these relationships imply that BRI was lower at sites with higher past SST variability and high past heat stress. The relationships between BRI and the 2009–2010 heat stress metrics were also negative – implying lower BRI with higher heat stress – but not significant. The relative abundance of massive *Porites* and *Porites rus* (RA_POR_) is also negatively correlated with BRI, though not significant using the Bonferroni correction.

**Table 1:** Linear regressions between 2009–2010 BRI and individual physical and biological variables.

Variable^a,b^			All sites		N Gilberts only	
			R	RMSE	R	RMSE
2009–2010 heat stress	MaxDHW_9–10_	Max weekly DHW	−0.365	0.116	−0.400*	0.013
	MaxSST_9–10_	Max weekly SST	−0.003	0.125	−0.290	0.014
	WkDHW4	# weeks DHW > 4	−0.287	0.119	−0.425*	0.013
	WkDHW8	# weeks DHW > 8	−0.371	0.116	−0.513**	0.012
	PAR	Boreal winter PAR	0.834**^,^^^	0.069	0.703**^,^^^	0.010
Past climate	CV_SST_	CV weekly SST	−0.599**^,^^	0.100	−0.734**^,^^^	0.010
	σ_SST_	σ, max monthly SST	−0.538**	0.105	−0.674**^,^^^	0.011
	σ_wk_	σ, max weekly SST	−0.463*	0.111	−0.652**^,^^^	0.011
	MaxSST_avg_	Mean, max weekly SST	−0.099	0.124	−0.314	0.014
	MMM	Max monthly mean SST	0.497**	0.108	0.175	0.014
	MaxDHW_avg_	Mean, max annual DHW	−0.634**^,^^^	0.096	−0.512**	0.012
	WkDHW4_avg_	Mean # weeks DHW > 4	−0.693**^,^^^	0.090	−0.493*	0.013
	WkDHW8_avg_	Mean # weeks DHW > 8	−0.530**	0.106	−0.474*	0.013
	fDHW4	Freq. years with DHW > 4	−0.738**^,^^^	0.084	−0.545**	0.012
	fDHW8	Freq. years with DHW > 8	−0.441*	0.112	−0.164	0.014
Other	Depth	Survey depth	0.171	0.123	−0.150	0.014
	Chl a	2009/10 chl a	−0.341	0.117	−0.401*	0.013
	Chl a (avg)	Long-term mean chl a	−0.377	0.115	−0.419*	0.013
	Expos	Exposure	−0.134	0.124	−0.434*	0.013
	Waveh	Wave height	0.552**	0.104	0.758**^,^^^	0.009
	RA_POR_	Relative abundance *Porites*	−0.521**	0.106	−0.596**^,^^	0.012
Position	Lat	Latitude (absolute value)	−0.450*	0.111	0.755**^,^^^	0.009
	Long	Longitude	0.751**^,^^^	0.082	−0.665**^,^^^	0.011

^a^^,^***p < 0.01, **p < 0.05, *p < 0.1.

^b^With Bonferroni correction ^^p < 0.01, ^p < 0.05.

If the southern Gilberts (Tamana and Tabiteuea) data, which were collected earlier in the year, are removed, the results are similar, with significant relationships between BRI and most metrics of past climate variability as well as wave height and RA_POR_. Additionally, if the analysis is repeated with BRI averaged across different depths at each site, there is no difference in the significance or direction of any of the regressions (not shown).

The individual metrics of past SST variability and past heat stress are significantly correlated with each other as well as with the 2009–2010 heat stress metrics and chlorophyll-a (chl a) concentration (Fig. S1), and significantly negatively correlated with PAR, significant wave height, and the absolute value of latitude. These relationships imply that the more equatorial sites (e.g., Tarawa and Abaiang) – where BRI was lower and past SST variability and chl a concentrations were higher – experienced greater heat stress during the 2009–2010 event and during previous years, but also lower incoming solar radiation (represented here by PAR) and lower wave heights during the 2009–2010 event.

Further analysis using generalized additive models suggests that reduced solar radiation and greater past temperature variability buffer heat stress experienced by Gilbert Islands coral reefs. A model employing only PAR and a metric of past heat stress frequency (fDHW4) as predictor variables explains 86.7% of the deviance (Table 2). Incorporating CV_SST_ and chl a significantly increases the fit according to an analysis of deviance, but the Akaike Information Criterion (AICc) remains the same (AICc = −80.5); incorporating other additional variables decreased AICc. Given the correlation between PAR and CV_SST_, a similar model (fDHW4, CV_SST_, chl a) that excluded PAR had higher AICc (−65.9) and explained less deviance (82.4%); a further model incorporating RA_POR_ explained the most deviance (89.2%).

**Table 2:** Results of generalized additive model (GAM) analysis to assess the relationship between BRI and remote sensing variables.

Model	r^z^ (adj)	Deviance explained	GCV	p-value	AICc
CV_SST_, fDHW4, Chl a, RA_POR_	0.862	0.893	0.002964	6.16E-12	−73.1606
PAR, fDHW4	0.856	0.867	0.002611	6.51E-13	−80.5355
**Northern Gilberts (only)**					
CV_SST_, fDHW4, Chl a, RA_POR_	0.633	0.668	0.002283	1.63E-10	−77.3076
PAR, fDHW4, Chl a, RA_POR_	0.644	0.722	0.002582	7.57E-10	−70.6817

This relationship is, however, influenced by the inclusion of the southern Gilbert Islands (Tamana, Tabiteuea) which experienced greater PAR and greater bleaching response. When only the northern Gilbert Island data are included in the analysis, the model excluding PAR (fDHW4, CV_SST_, chl a, RA_POR_) has the lowest AICc, although this model explains less deviance than one in which PAR replaces CV_SST_ (Table 2). Model rankings were the same employing a thin rather than cubic spline, with data averaged across depth at each survey site, and using the same generalized linear model (GLM) formulations (not shown). However, a GLM incorporating interactive effects (PAR, fDHW4, PAR × fDHW4) across all sites had the lowest AICc and explained the most deviance (83.5%).

### Changes in coral and benthic communities after 2010

There were inconsistent changes in benthic cover and coral community composition between 2010 and 2012 across South Tarawa, Abaiang, and Butaritari based on the nine sites surveyed in both years (Table S5). At 10–12 m depth, live hard coral cover declined across seven of the nine sites from a mean of 37.6% in 2010 to a mean of 33.4% in 2012, but the change was not significant (p = 0.525, Welch’s t = 0.650; see Materials and methods section). The only taxa that exhibited significant changes across all sites were all relatively uncommon (<2% coral cover; Table S5). Within atolls, only *Pocillopora* in Abaiang (from 4.6–9.9% in 2010 to 7.5–14.6% in 2012) and *Heliopora* in South Tarawa (2.1–6.3% to 6.7–14.8%) exhibited consistent increases from 2010 to 2012 between years at all sites. The results are similar with the shallow data (not shown).

**Figure 3:**
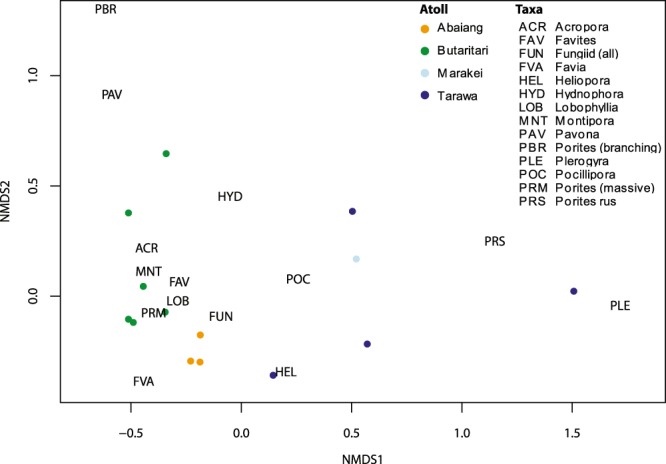
Non-metric multidimensional scaling using Bray-Curtis (BC) dissimilarities plot of coral community composition at 10–12 m depth sites from 2012 surveys (stress = 0.089). Coral genera that comprised <0.5% of observations are excluded.

**Figure 4:**
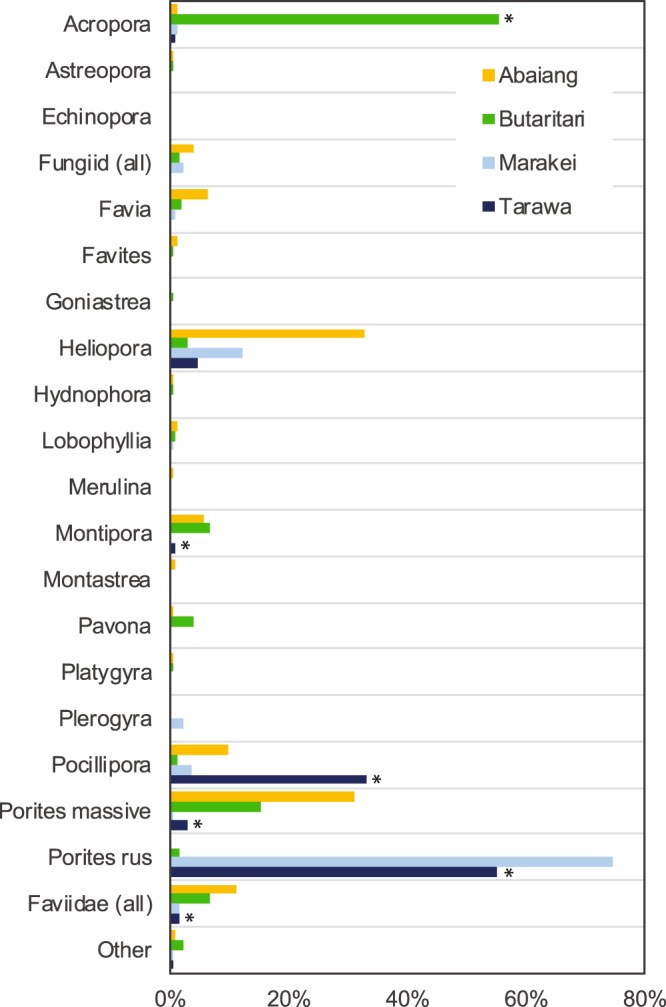
Composition of coral community by atoll in 2012 (10–12 m surveys). Asterisk indicates significant difference (p < 0.05) from other atolls.

The 2012 survey data at 10–12 m depth, which covered the greatest range of sites (n = 14), indicate that there were more distinct differences in benthic cover and coral community composition between the South Tarawa, Abaiang, and Butaritari sites than between sites within any of the atolls. Among the major benthic categories, live hard coral cover was higher in South Tarawa (mean = 49.4%; p = 0.114, permutation test) and Butaritari (mean = 37.5%; p = 0.0352, Welch’s t = −2.678) than in Abaiang (mean = 21.9%). The differences in coral community composition between the three atolls is illustrated with non-metric multidimensional scaling (NMDS) conducted with Bray-Curtis (BC) dissimilarities using coral categories (Fig. 3; stress = 0.089). The South Tarawa sites, which are subject to the most human disturbance, have the most distinct coral community; the BC values for coral community composition between South Tarawa sites and all other sites ranged from 0.45 to 0.99 (mean = 0.85), whereas the BC values within the Tarawa sites ranged from 0.15 to 0.69 (mean = 0.35). This is driven primarily by the dominance of *Porites rus* in Tarawa (mean = 74.5% of coral cover), but also by the lack of taxa otherwise common throughout the Gilbert Islands like *Acropora* (mean = 1.3%), massive *Porites* (mean = 0.5%), *Montipora* (mean = 0.1%), and members of the *Faviidae* family, all of which were significantly lower at Tarawa than the other atolls (Fig. 4). Community composition at the single Marakei site, located close to the port and the main center of human habitation, is most similar to the South Tarawa sites due to the dominance of *Porites rus* (55.2%).

Conversely, the Butaritari sites were dominated by *Acropora* (55.3% of coral cover), with significantly higher cover than at Abaiang (1.2%; p < 0.001, Welch’s t = −10.561) and Tarawa (1.3%; p < 0.0001; Welch’s t = 10.556). The Butaritari sites also had a larger number of genera present (n = 12–20 vs. n = 5–14 in Tarawa, n = 5–9 excluding TRW2). There is also greater dissimilarity between the Butaritari and Abaiang sites than between the sites within each of those atolls (Fig. 3). This is driven by the significantly greater presence of *Heliopora*, massive *Porites*, and *Pocillopora* in Abaiang, as well as the lack of *Acropora* (Fig. 4). The results are similar in the shallow data (not shown).

The fraction of coral in common categories that was living in the 2012 surveys further illustrates differences between atolls and the trajectory of coral cover (Table S6). For example, in South Tarawa, 57.0% of all coral cover, including 96% of the dominant taxa *Porites rus*, was living. Qualitative observations found that live *Porites rus* was overgrowing dead colonies of a historically more diverse coral community in South Tarawa, including very large massive *Porites* colonies and *Faviidae*. Conversely, in Abaiang, 41.4% of all coral cover was living, including 73.5% of *Heliopora* and 60.5% of *Pocillipora* colonies.

### Drivers of benthic community composition

**Figure 5:**
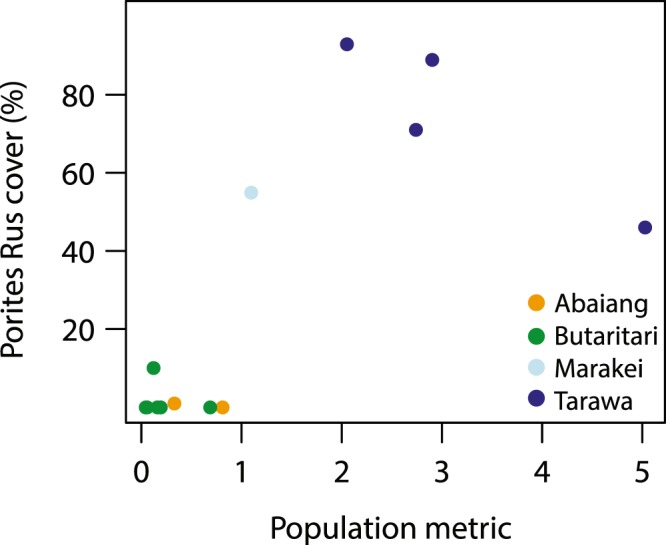
*Porites rus* as a percent of all live coral cover vs. the population metric, based on surveys collected at 10–12 m depth in 2012. The population metric was computed for each field site by dividing the population of the closest village(s) by the distance along the coastline to the center of the village(s).

To assess the drivers of differences in community composition across sites, permutational-based multivariate analysis (PERMANOVA^20^) was conducted with the 2012 survey data using atoll, the population metric, and three of the environmental variables (CV_SST_, fDHW4, chl a), selected based on the generalized additive model (GAM) evaluation. The analysis found significant differences in the benthic community composition at 10–12 m depth based on atoll (p = 0.001) and the population metric (p = 0.001). If repeated without atoll as a factor, given its correlation via latitude with most environmental variables, there are significant differences in the benthic community composition at 10–12 m depth based on the population metric (p = 0.002) and CV_SST_ (p = 0.040), as well as a weaker relationship with fDHW4 (Table 3). The findings are similar if repeated using only the coral community composition and/or both the 3–5 m depth and 10–12 m depth survey data (not shown); after atoll, the relationship with the population metric is strongest in each case (p ≤ 0.001), followed by that with CV_SST_. The relationship between atoll, population, and the coral community composition is illustrated by contrasting the population metric with the coral community composed of *Porites rus* (Fig. 5). *Porites rus* dominates the coral community in the heavily populated South Tarawa sites as well as the Marakei town site but is largely absent at other sites.

**Table 3:** PERMANOVA of drivers of benthic composition, based on 2012 surveys at 10–12 m depth.

Variable	Df	Sums of Sqs	Mean Sqs	F (model)	r^2^	p-value
Population	1	0.6072	0.6072	5.5621	0.2807	0.002
fDHW4	1	0.2474	0.2474	2.2659	0.1144	0.076
CV_SST_	1	0.2914	0.2914	2.6693	0.1347	0.040
Chl a	1	0.0348	0.0348	0.3185	0.0161	0.899
Residuals	9	0.9826	0.1092	0.4542		
Total	13	2.1634	1

## Discussion

The available survey data suggest that the outer reef coral communities in the central Gilbert Islands have been relatively resilient to repeat heat stress associated with Central Pacific El Niño events. The analyses presented here suggest that past climate experience and local human disturbance have together influenced the observed variability in bleaching response and coral community structure across the region. First, sites in the more equatorial reefs, which are exposed to ENSO-driven year-to-year variability in climate, show evidence of acclimation to heat stress. As a consequence, heat stress is found to be a poor predictor of the variability in bleaching intensity within the region. Second, sites exposed to local human disturbance have coral communities that are dominated by bleaching-resistant species. At the South Tarawa sites, the combination of ENSO-driven climate variability and local human disturbance potentially drove a positive feedback effect that promotes a low-diversity community dominated by weedy species (*Porites rus*). We explore the interaction between climate variability and local disturbance below.

### Climate variability and reef resilience

The results support past evidence, based on cores collected during the 2010 surveys employed in this study, that massive *Porites* in Tarawa and Abaiang were more bleaching-resistant than those in more off-equatorial Butaritari despite experiencing higher DHW values^15^. That response was attributed to the individuals at the more equatorial sites having experienced higher inter-annual SST variability and hence having persisted and acclimatized through more severe past heat stress. Here, analysis of the coral community data from 2010 also finds that past heat stress is a better predictor of bleaching response than the DHW values.

However, the examination of a broader suite of physical variables in this study suggests that lower incoming light levels (represented here by PAR) at the more equatorial sites during heat stress events may explain some of the pattern in bleaching response, and potentially contribute to the observed bleaching resistance described in Carilli *et al*.^15^. Coral bleaching is caused by the synergistic effect of elevated light and temperature, which together cause breakdown of normal symbiont photosynthetic pathways and cause host and symbiont cellular damage^21^. Reduced incoming light due to cloudy conditions has been shown to mitigate bleaching elsewhere in the Pacific^22^. The central equatorial Pacific typically experiences deep convective activity and high cloudiness during El Niño events, evidenced by the low PAR values in Tarawa and Abaiang during the 2009–2010 event. Reduced incoming light due to cloudiness also occurred during the 2004–2005 event; according to the available Kiribati Meteorological Service data, Tarawa experienced only 2.2 hrs/day of clear skies from November 2004 through March 2005, one-third of the long-term average (6.2 hrs/day).

There are several important caveats to the possible role of reduced incoming light due to cloudiness in mitigating bleaching. First, due to the narrow latitudinal range of the SEC, the influence of ENSO on winds, currents, and upwelling strength, as well as PAR, also decrease with latitude in the central equatorial Pacific. Therefore, models for 2010 BRI that excluded PAR are also significant and feature low AICc. Second, the strength of PAR specifically as a predictor could be at least partially an artefact of the timing of the surveys, a critical issue too often ignored in bleaching studies. The southern Gilberts sites, which featured the highest PAR and highest BRI values, were surveyed earlier in the year; corals that were bleached but later recovered could have been recorded as bleached at the southern sites but not the northern sites. When these sites are excluded from the analysis, the model excluding PAR had the lowest AICc. Finally, this analysis is conducted with high resolution satellite-derived data products which can have retrieval issues and represent surface rather than reef conditions (e.g., do not capture light attenuation).

Despite uncertainty about the precise role of individual variables like light or past temperature variability, these findings clearly indicate that the DHW metric can be a poor predictor of the variability in bleaching response within this region. This finding echoes a number of recent studies testing the use of alternative temperature variability metrics and other environmental variables to predict the occurrence or intensity of bleaching^12,23–25^. The utility of DHW or similar simple threshold-based metrics for bleaching prediction may be a function of scale. The DHW approach may be broadly effective at predicting the likelihood of some bleaching across a region; NOAA’s DHW-based Bleaching Alert did correctly predict the events described in this study. The same approach may, however, be ill-suited to high-resolution prediction of bleaching intensity in regions like the central equatorial Pacific that feature sharp geographic gradients in key variables, like background temperature variability, cloudiness, and local stressors, that influence bleaching susceptibility and coral community structure.

### Resilience of disturbed reefs

Coral cover was highest and most stable over time at the South Tarawa sites (e.g., mean of 49.4% coral cover in 2012) where the weedy^26^ species *Porites rus* dominated the coral community. Home to roughly half of the Kiribati population, South Tarawa experiences high nearshore fishing pressure due to the population density and also high nutrient pollution due to limited access to toilets, limited sewage treatment, and poor overall waste management^18^. Construction of solid causeways linking the islets in South Tarawa have contributed to decline of water quality and altered sediment deposition patterns by limiting exchange between the lagoon and open ocean^27^.

The combination of local stressors and historical heat stress in South Tarawa may have promoted the spread of *Porites rus* over time and limited the survival of other coral species. *Porites rus* has been shown to tolerate heat stress^9,28–30^, nutrient loading^31,32^, and turbid environments^33^, possibly due to thick tissues that may buffer environmental stress^34^ and its ability to grow in plating or branching forms depending on the environmental conditions^35^. As a brooding species, *Porites rus* may also be more likely to recover from partial mortality and spread into large stands (visible at sites TRW08, TRW10, TRW11, and MRK01). The loss of more bleaching-sensitive taxa, like *Pocillopora spp*., during past heat stress events^14^ likely offered an expansion opportunity for species like *Porites rus* that can adapt to stressed South Tarawa reefs. Observations from the year 2000 indicate *Porites rus* was likely common across some South Tarawa outer reefs, but so were other currently rare taxa^36^. The dead coral data in this study suggest that *Porites rus* expanded into space previously occupied by *Pocillipora*, *Lobophyllia*, *Acropora*, and other taxa lost due to bleaching.

The differences in the composition of the coral community between the other atolls and South Tarawa may be both a consequence of historical heat stress and a driver of the response to the 2009–2010 heat stress. By 2012, coral cover was generally lowest at the Abaiang sites (mean of 21.9%), despite Abaiang experiencing less fishing pressure, nutrient and sediment loading, and other local impacts than neighbouring South Tarawa. Coral cover declined at the Abaiang sites after the 2004–2005 heat stress due in large part to the loss of bleaching-susceptible *Pocillopora*. In the 2010 and 2012 surveys, the Abaiang coral community was increasingly dominated by more heat-resistant taxa including the octocoral *Heliopora coerulea* and massive *Porites*. *Heliopora coerulea* is regionally uncommon but has been found to expand after mass bleaching events elsewhere in the Indo-Pacific^37–39^.

By contrast, Butaritari, subject to less frequent and less severe heat stress than Abaiang and Tarawa, had the only sites in which bleaching-susceptible *Acropora* was dominant; bleaching of *Acropora* was the primary driver of the higher 2010 BRI values at Butaritari (compared to Tarawa and Abaiang). *Acropora* was uncommon at Abaiang and Tarawa sites even before the 2004–2005 bleaching event^14,36^. Exposure was not a driver of the recorded pattern in coral community structure, in part because of the dominance of *Porites rus* at both more leeward and more windward sites in South Tarawa as well as at the protected Marakei site. Data from difficult-to-access windward coasts of Abaiang and Butaritari would be necessary to fully test the role of exposure.

There are a variety of mechanisms by which the Gilbert Islands’ coral reefs may be adapting or acclimating to repeat heat stress, including the community shifts described here, as well as physiological acclimatization, shuffling, or shifting of the *Symbiodinium* community, and directional selection. The available data provides some evidence for physiological acclimatization of corals in Tarawa and Abaiang. Tissue samples collected from massive *Porites* after the 2009–2010 heat stress event found that Tarawa and Abaiang corals had thicker tissues and significantly higher levels of triacylglycerol, a lipid quickly metabolized during periods of stress, than Butaritari corals^15^. Although no data for the *Symbiodinium* community is available for 2010, samples collected in 2009 (see Additional sources of data, in Materials and methods section) showed that *Symbiodinium* clade C dominated 87% of colonies in Tarawa and Abaiang^40^. Samples from *Porites rus* and massive *Porites* (*Porites lutea* and *Porites lobata*) contained only Type C15^40^. Though limited, the data supports evidence from French Polynesia that the ability of *Porites* species, particularly *Porites rus*, to adapt to relatively inhospitable environments is driven by phenotypic plasticity rather than changes in the *Symbiodinium* community^35^.

An important caveat is that the benthic composition in parts of South Tarawa could also be influenced by elevated trace metal concentrations. The site TRW10, for example, features corallimorphs, cyanobacteria, and low crustose coralline algae cover, similar to “black reefs” reported elsewhere in the Pacific^41^. The benthic composition of such sites in Kiribati’s Line Islands^42^ and Phoenix Islands^43^ have been tentatively linked to iron leaching from shipwrecks. Although there were no large shipwrecks on South Tarawa’s outer reef at the time of these surveys, there are several likely sources of trace metal inputs to this ecosystem over the past seventy years. First, the lagoon side of Betio was the site of a World War II battle^19^ and continues to feature wreckage from that battle and from more recently abandoned ships. Before the closure of a causeway between Betio and Bairiki in the early 1970s, lagoon flushing would have brought leached metals to outer reef sites like TRW10. Second, landfills, built on reclaimed land on the lagoon side, seep leachates into the lagoon and are often penetrated by high tides^17^. Third, until a foreign scrap metal buyer emerged in the mid-2000s, used vehicles, mechanical equipment, and other metallic refuse regularly sat abandoned on the beaches of South Tarawa^17^. Further surveys and water quality analyses would be necessary to properly test the black reef hypothesis.

### Climate refugia: The resilient reef or the desirable reef ?

Identifying climate refugia may be critical to the long-term survival of coral reefs given the ongoing threat of rising carbon dioxide concentrations and climate change. The coral reefs in the Gilbert Islands may qualify as climate refugia due to a globally unique climate experience, including little seasonal temperature variation but high year-to-year variability in SST, heat stress, and cloudiness. Coral reefs to the west (e.g., Palau) similarly experience limited seasonality, but not the same magnitude or frequency of ENSO-driven SST variability^12^. Only the few equatorial islands and atolls to the east – the U.S. Pacific territories (Jarvis, Howland, and Baker), and parts of Kiribati’s Phoenix Islands and Kiribati’s Line Islands – are influenced enough by the SEC to experience marine climate variability similar to that of the Gilbert Islands. In addition, the western sides of the equatorial atolls experience upwelling of cooler waters due to the interaction of the islands and the eastward flowing Equatorial Undercurrent (EUC), a nutrient rich countercurrent found at 50–200 m depth^44^. Although this effect may be limited during El Niño-driven heat stress events, when the EUC is typically weakened, it may partially offset heat stress associated with climate change^44^. For these reasons, other studies have also recently proposed the central equatorial Pacific^45^ and the Gilbert Islands in particular^44^ as potential climate refugia.

The results of this study support the notion that this climate experience may be creating resistance to, and resilience from, recent bleaching-level heat stress in the central Gilbert Islands. The results, however, also suggest that resilience may come at the expense of ecosystem services. Highly disturbed sites in South Tarawa are resilient to bleaching because of the dominance of a single weedy coral species, *Porites rus*. These sites feature not only low coral diversity but also low structural complexity, which limits habitat for other reef organisms and, most critically for a country threatened by sea-level rise, limits wave attenuation. The coral reefs may be resilient from a climate perspective but may not be desirable from an ecological or societal perspective.

The resilience of unmanaged, low diversity reefs raises questions for developing nations like Kiribati about setting management and protection priorities as the climate warms. The classic approach to conservation was to focus on the most pristine and diverse systems. More recent research points to the importance of identifying and protecting naturally resilient systems^46^, which may or may not feature high diversity. South Tarawa is a clear case of the latter. Yet if resources for coastal management are limited, do you focus them on reefs that already maintain coral cover despite numerous local stressors and multiple heat stress events? Resources may be better apportioned to managing or protecting reefs in places like Abaiang or even Butaritari, which display less resilience to heat stress than South Tarawa but are more diverse and likely more resilient than reefs in other parts of the Pacific. As in the fairy tale *Goldilocks and the Three Bears*, it may be best to focus effort on the middle reefs that are neither too pristine nor too naturally resilient.

## Materials and Methods

### Site history and description

The Gilbert Islands are primarily coral atolls consisting of an outer rim of narrow islets surrounding a lagoon, although the southern part of the chain includes atolls with limited lagoons (Tabiteuea) and low-lying reef islands (Tamana). The outer reef perimeter comprises the majority of modern coral reef development. Lagoon reefs are generally limited to isolated patches and exposed channels due to turbid conditions in shallow and inshore areas. The islands were first settled by people from Melanesia over one thousand years ago^47^. In the late 20^th^ century, population growth and the demands of the cash economy began spurring migration from outer atolls where subsistence practices dominate to the administrative centres in South Tarawa^18^. Before 2005, benthic surveys were limited to isolated studies of easily accessible lagoon patch reefs and nearshore reefs in Tarawa, often conducted through externally-funded marine resources or geological assessments^36,48,49^. Between 2005 and 2009, the lead author worked with Kiribati’s Ministry of Fisheries and Marine Resource Development (MFRMD) to develop a training and monitoring program and to conduct, via free-diving, repeat shallow photo-quadrat surveys at five sites in Tarawa and Abaiang^14^.

This study draws on data from subsequent SCUBA-based surveys that were carried out in May 2010 and May 2012 in conjunction with MFMRD work (Fig. 1; full list of sites in Table S3). All field work and data usage was approved by the MFMRD following Kiribati government guidelines and policy. Sites were selected either based on the availability of data from previous surveys, or initial manta tows used to identify the variance in total coral, algae, and dead coral cover. Several sites were not surveyed in both 2010 and 2012 due to weather (e.g., TRW05 is only accessible in calm conditions), logistics (e.g., local boat availability in Butaritari), and health (e.g., dengue fever outbreak limited the 2012 North Tarawa/Abaiang surveys). The location of site TRW08 was shifted in 2012 for safety reasons; the original site lay dangerously close to the sewage outflow from the Tarawa hospital. One opportunistic site close to the main village and small boat landing in Marakei Atoll was included in the 2012 survey because of a stop made by the MFMRD vessel in transit to Butaritari. The data from Tamana and Tabiteuea comes from video collected by the MFMRD in January 2010, in consultation with the lead author^50^.

### Survey protocol

In 2010 and 2012, photo-quadrat surveys and coral size distribution were conducted along a haphazardly laid 50 m transect at 3–5 m and at 10–12 m depth (where physically possible). Photos were taken on alternating sides of the transect line using a 0.83 m^2^ quadrat or equivalent markings made along the transect line. The sampling design allows for five replicate 10 m transects at each depth, but for the purposes of this study, benthic cover was computed based on the entire transect (see Data Analysis below).

For each transect, 25 photos were employed in the analysis, representing 20 m^2^ of benthos. Benthic cover was manually recorded at 50 points in each image leading to 1,250 points per transect. Live coral cover was generally recorded at the genus level; *Porites* were categorized as branching, massive, and *Porites rus*, the encrusting species common at several sites. The octocoral *Heliopora coerulea*, common in Kiribati, is also included in the list of coral genera. Bleached corals, recently dead corals (e.g., post-bleaching), dead corals covered in turf algae (<~2 cm thick), and dead coral covered in crustose coralline algae were identified to the genus level where possible. Earlier shallow photo-quadrat surveys conducted at five sites in each of Tarawa and Abaiang in 2005 and 2009^14^ were also reanalysed following this protocol.

The number of images necessary to capture the variance in coral cover at each site was determined using a Monte Carlo analysis of point observations from the 2010 survey. Benthic cover was manually recorded at 50 random points in images from one site in each of Tarawa, Abaiang, and Butaritari, using the CPCe software package. For n = 1 to n = 50, percent cover of all live coral and of the four most common coral taxa at each site was calculated 1,000 times using a random sampling of n images. The analysis showed the coefficient of variation of the 1,000 estimates of the percent cover categories began to asymptote with n > 20 images and decreased below 0.25 with n > 25 images.

### Bleaching Response Index (BRI)

The bleaching response at each site and depth in the 2005 and 2010 field data was estimated using BRI, following McClanahan^29^. The percent of individuals in each of seven categories of bleaching intensity (c_1_ = normal, c_2_ = pale, c_3_ = 0–20%, c_4_ = 20–50%, c_5_ = 50–80%, c_6_ = 80–100% bleached, c_7_ = recently dead) was used to compute a BRI by taxa, and for all corals, according to:

$$BRI=\frac{(0{c}_{o}+1{c}_{1}+2{c}_{2}+3{c}_{3}+4{c}_{4}+5{c}_{5}+6{c}_{6}+7{c}_{7})}{6}$$

Use of the BRI enabled comparison of bleaching response from both photo-quadrat surveys conducted as part of this research and videos collected via haphazard swim during the 2010 assessments at Tamana Island and Tabiteuea Atoll^50^.

### Environmental variables from remote sensing data

A suite of physical and chemical variables was extracted for each field site from remotely sensed data for comparison to BRI. The weekly SST and DHW (in °C-week) for 1985–2012 were obtained from Coral Reef Watch data^51^. These data were used to compute metrics of the 2009–2010 heat stress: maximum SST from the 2009–2010 event (MaxSST_9–10_), maximum DHW from the 2009–2010 event (MaxDHW_9–10_) and the number of weeks with DHW > 4 °C-week and DHW > 8 °C-week (WkDHW4, WkDHW8). We also calculated various metrics of pre-event climate variability (1985–2009), proposed by previous studies^12^: coefficient of variation of weekly SSTs (CV_SST_), standard deviation of monthly SST from the warmest week (σ_wk_) and month of each year (σ_mon_), mean of the maximum weekly SST from each year (MaxSST_avg_), the maximum monthly mean (MMM), the mean maximum annual DHW from all years (MaxDHW_avg_), mean number of weeks with DHW > 4 °C-week and DHW > 8 °C-week (WkDHW4_avg_, WkDHW8_avg_) and the frequency of years with DHW > 4 °C-week and DHW > 8 °C-week (fDHW4, fDHW8).

In addition, PAR, used here as a proxy for incoming solar radiation, and the chlorophyll-a concentration (chl a) for each site were obtained for 2009–2010 boreal winter from 4 km resolution NASA’s MODerate Imaging Spectroradiometer (MODIS) data, available at: http://oceancolor.gsfc.nasa.gov; long-term mean chlorophyll a (chl a_avg_) for 2000–2010 was also computed. Significant wave height (waveh, in m) for the 2009–2010 boreal winter was obtained from NOAA WaveWatch III model data, available at http://polar.ncep.noaa.gov/waves/download.shtml. Exposure (expos) of each site to prevailing winds and waves was also roughly estimated on a four-point scale, based on the site location relative to land.

### Additional sources of data

Nearby human population was used as a proxy for the level of local human disturbance to coral reefs. A population metric was computed for each field site by dividing the population of the closest village(s) by the distance along the coastline to the center of the village(s), following Carilli and Walsh^52^. The metric was standardized based on the average across all the field sites.

Data on the diversity of algal symbionts (genus *Symbiodinium*) from a May 2009 survey^40^ of Abaiang and Tarawa is discussed for context. Replicate (n = 5) tissue samples of common coral species were collected haphazardly at between 5–10 m depth. Fragments of roughly 2 cm length were collected from living coral colonies using a chisel with branching colonies or a hammer and chisel on massive and encrusting colonies. The samples were brought onto the boat and placed in 2 ml labelled vials and preserved with saline dimethyl sulfoxide. The presence of different *Symbiodinium* types in individual coral species was identified using standard methods by the Baker lab at the University of Miami.

### Data analysis

All analyses were conducted in R version 3.2.5. Differences in benthic cover, heat stress metrics, and BRI were tested between atolls and between years using the different sites that were consistently monitored within each atoll as replicates. All data were first tested for normality using the Shapiro-Wilk test. We used Welch’s t-test to test for significant differences between years and atolls when data were normal and permutation tests when data were not normal; when a p-value but no t-value is reported in the text, the test was a permutation test. The differences in coral communities between years and sites was also analysed using an NMDS based on BC dissimilarities of each taxa’s abundances per site, using vegan package version 2.4–3^53^. Analysis was conducted using only categories that represent >0.5% of coral cover across all sites in the interest of readability of the NMDS; a sensitivity test using all categories exhibited similar results (mean difference in BC dissimilarities <1%).

The relationship between 2010 BRI across all available sites at both depths and each of the environmental variables (plus latitude, longitude, and the relative abundance of massive *Porites* and *Porites rus*) was initially evaluated using simple least squares regression. Critical values for all tests were adjusted using the Bonferroni correction to avoid Type I errors across multiple comparison tests. Models for BRI and the remote sensing variables, excluding significantly correlated predictor variables, were then fit using the GAM function in R^54^. The cubic spline with shrinkage was used and smoothers were restricted to three or fewer to minimize model overfitting. The process was repeated employing fewer predictor variables and compared using the second-order AICc and an analysis of deviance table (computed using the F-test). The best models were also evaluated against GLMs in order to include possible interactive effects. The process was conducted using surveys at different depths from each site as independent data points and using combined results across all depths from each site.

Finally, the influence of environmental variables and human disturbance, represented by the population metric, on the structure of the benthic community across sites was assessed via PERMANOVA^20^ using vegan package version 2.4–2^55^. This analysis was conducted with the 2012 survey data, which included the largest number of sites, using all benthic cover categories and also repeated using only coral taxa to specifically examine drivers of coral community composition.

## Supplementary information

Supplementary Material

## Data Availability

All data (benthic data and environmental variables for each field site) employed in this study will be available at http://www.simondonner.com or by contacting the corresponding author.
