# Supplementary material for: Resilience of Central Pacific reefs subject to frequent heat stress and human disturbance
Source: Sci Rep. 2019 Mar 5;9:3484. doi: 10.1038/s41598-019-40150-3 (PMC6401028; doi:10.1038/s41598-019-40150-3)
Supplement: Supplementary file 1 — Supplementary Material [file 41598_2019_40150_MOESM1_ESM.docx]

**SUPPLEMENTARY MATERIAL**

**Resilience of Central Pacific reefs subject to frequent heat stress and human disturbance**

**Authors:** Simon D. Donner (simon.donner@ubc.ca)^1^, Jessica Carilli^2^

**Affiliations:**

^1^ Department of Geography, 1984 West Mall University of British Columbia, Vancouver, British Columbia, Canada V6T 1Z2

^2^ Australian Nuclear Science and Technology Organization, New Illawarra Rd, Lucas Heights, NSW, Australia 2234

Now at: Energy and Environmental Sciences, Space and Naval Warfare Systems Center Pacific, 53475 Strothe Rd. San Diego, CA 92152 USA

**Donner and Carilli – Supplementary Material**

The Supplementary Material includes six tables and one figure.

**Table S1. Bleaching Response Index for common taxa at five sites with observations in the 2004-2005 and 2009-2010 heat stress events**

| SITE | All taxa^*^ | | *Acropora*^*^ | | *Pocillipora*^*^ | | *Porites* (massive)^*^ | | *Montipora* | |
| --- | --- | --- | --- | --- | --- | --- | --- | --- | --- | --- |
|  | 2005 | 2010 | 2005 | 2010 | 2005 | 2010 | 2005 | 2010 | 2005 | 2010 |
| ABG01 | 0.40 | 0.03 | 0.75 | 0.00 | 0.89 | 0.06 | 0.02 | 0.00 | 0.04 | 0.04 |
| ABG02 | 0.53 | 0.03 | 0.95 | 0.05 | 0.97 | 0.00 | 0.21 | 0.00 | 0.12 | 0.07 |
| ABG03 | 0.34 | 0.07 | 1.00 | 0.50 | 0.98 | 0.00 | 0.12 | 0.06 | 0.11 | 0.12 |
| TRW02 | 0.63 | 0.06 | 0.95 | 0.13 | 0.89 | 0.00 | 0.54 | 0.00 | 0.53 | 0.00 |
| TRW08 | 0.24 | 0.04 | 0.00 | 0.00 | 0.52 | 0.11 | 0.00 | 0.00 | 0.21 | 0.14 |

^*^Significantly different (p<0.05) between years

**Table S2. Comparison of 2004-2005 and 2009-2010 thermal stress events**

| SITE^1^ | 2004-2005 Stress event | | | 2009-2010 Stress event | | |
| --- | --- | --- | --- | --- | --- | --- |
|  | Max DHW (°C-week) | Weeks of Alert  Level I Level II | | Max DHW (°C-week) | Weeks of Alert  Level I Level II | |
| ABG01^*^ | 18.8 | 25 | 21 | 15.4 | 21 | 13 |
| ABG02^*^ | 5.7 | 11 | 0 | 6.4 | 11 | 0 |
| ABG03^*^ | 18.3 | 22 | 17 | 16.3 | 19 | 12 |
| BUT01 | 13.0 | 19 | 10 | 8.1 | 12 | 1 |
| BUT02 | 5.9 | 9 | 0 | 7.7 | 12 | 0 |
| BUT03 | 5.9 | 9 | 0 | 7.7 | 12 | 0 |
| BUT04 | 11.8 | 20 | 11 | 10.4 | 14 | 6 |
| BUT05 | 8.8 | 14 | 1 | 4.7 | 9 | 0 |
| BUT06 | 9.3 | 15 | 2 | 3.7 | 0 | 0 |
| BUT08 | 11.3 | 16 | 12 | 8.8 | 12 | 1 |
| BUT09 | 11.3 | 16 | 12 | 8.8 | 12 | 1 |
| MRK01 | 12.9 | 17 | 11 | 16.3 | 21 | 14 |
| TRWN05 | 13.8 | 23 | 12 | 15.7 | 19 | 14 |
| TRWN07 | 16.8 | 25 | 19 | 7.9 | 13 | 0 |
| TRW02^*^ | 15.8 | 29 | 17 | 13.9 | 19 | 12 |
| TRW08^*^ | 17.6 | 22 | 16 | 9.4 | 14 | 5 |
| TRW10 | 18.7 | 41 | 19 | 13.2 | 19 | 12 |
| TRW11 | 13.7 | 21 | 14 | 16.4 | 21 | 14 |

^*^Legacy sites with shallow-water surveys in 2005 and 2009

**Table S3: Benthic surveys by year and site, with hard coral cover at 3-5 m and 10-12 m depth (second row, if available)**

| **ATOLL** | **SITE** | **CODE** | **2000^1^** | **2004** | **2005** | **2009** | **2010** | **2012** |
| --- | --- | --- | --- | --- | --- | --- | --- | --- |
| **ABAIANG** | Manra | ABG01 |  |  | 21% | 43% | 31% | 20% |
|  |  |  |  |  |  |  | 28% | 25% |
|  | Nuotea | ABG02 | 37% |  | 23% | 21% | 22% | 26% |
|  |  |  | 28% |  |  |  | 18% | 14% |
|  | Tebonte-bike | ABG03 | 45% |  | 30% | 38% | 35% | 43% |
|  |  |  | 72% |  |  |  | 30% | 27% |
| **BUTARI-TARI** | South pass | BUT01 |  |  |  |  | 32% | 36% |
|  | Ocean tip | BUT02 |  |  |  |  |  | 42% |
|  |  |  |  |  |  |  |  | 42% |
|  | SW reef | BUT03 |  |  |  |  | 26% | 29% |
|  |  |  |  |  |  |  | 59% | 52% |
|  | Bikati | BUT04 |  |  |  |  |  | 43% |
|  |  |  |  |  |  |  |  | 26% |
|  | Western | BUT05 |  |  |  |  | 38% | 37% |
|  |  |  |  |  |  |  | 46% | 25% |
|  | Pearl Farm | BUT06 |  |  |  |  | 30% |  |
|  | Central pass | BUT08 |  |  |  |  | 41% | 53% |
|  |  |  |  |  |  |  | 36% | 44% |
|  | Tukurere | BUT09 |  |  |  |  | 26% |  |
| **MARAKEI** | Port | MRK01 |  |  |  |  |  | 17% |
|  |  |  |  |  |  |  |  | 16% |
| **NORTH TARAWA** | Na'a (N Tarawa) | TRWN05^2^ |  | 53% |  | 16% |  |  |
|  |  |  |  |  |  |  |  |  |
|  | NW Reef (N Tarawa) | TRWN07^2^ | 57% |  |  |  | 30% |  |
|  |  |  | 41% |  |  |  | 43% |  |
| **SOUTH TARAWA** | Teaoraereke | TRW02 |  | 21% | 11% | 8% | 14% | 11% |
|  |  |  |  |  |  |  | 32% | 25% |
|  | Bikenibeu | TRW08 | 7% | 20% | 20% | 40% | 34% | 19% |
|  |  |  | 28% |  |  |  |  | 60% |
|  | Causeway | TRW10 | 33% |  |  |  | 39% | 49% |
|  |  |  | 56% |  |  |  | 58% | 53% |
|  | Eita | TRW11 |  |  |  |  |  | 24% |
|  |  |  |  |  |  |  |  | 60% |

^1^ LIT data from Lovell (2000)

^2^ Quadrats not completed in 2012 due to weather and illness

**Table S4. 2009-10 Bleaching Response Index (common categories)**

| Site | Hard Coral | *Acropora* | *Faviidae* | *Montipora* | *Pavona* | *Pocillipora* | *Porites* | |
| --- | --- | --- | --- | --- | --- | --- | --- | --- |
|  |  |  |  |  |  |  | Massive | *P. rus* |
| ABG01S | 0.03 | 0.00 | 0.00 | 0.04 | - | 0.06 | 0.00 | - |
| ABG01D | 0.08 | 0.30 | 0.18 | 0.00 | 0.00 | 0.06 | 0.02 | - |
| ABG02S | 0.03 | 0.05 | 0.05 | 0.07 | - | 0.00 | 0.00 | - |
| ABG02D | 0.08 | 0.00 | 0.16 | 0.31 | 0.00 | 0.00 | 0.00 | - |
| ABG03S | 0.07 | 0.50 | 0.10 | 0.12 | 0.00 | 0.00 | 0.06 | - |
| ABG03D | 0.06 | 1.00 | 0.15 | 0.00 | 0.17 | 0.00 | 0.00 | - |
| ABG10S | 0.06 | 0.29 | 0.10 | 0.04 | - | 0.00 | 0.00 | - |
| ABG11S | 0.09 | 0.28 | 0.09 | 0.34 | 0.21 | 0.32 | 0.04 | - |
| BUT01 | 0.27 | 0.35 | 0.17 | 0.00 | 0.00 | 0.30 | 0.00 | 0.00 |
| BUT03S | 0.07 | 0.10 | 0.08 | 0.00 | 0.00 | 0.00 | 0.00 | - |
| BUT03D | 0.13 | 0.16 | 0.10 | 0.00 | - | 0.13 | 0.01 | - |
| BUT05S | 0.18 | 0.41 | 0.02 | 0.00 | - | 1.00 | 0.00 | - |
| BUT05D | 0.08 | 0.18 | 0.14 | 0.00 | 0.00 | - | 0.00 | - |
| BUT06S | 0.28 | 0.29 | 0.00 | 0.00 | - | - | 0.00 | - |
| BUT08S | 0.18 | 0.33 | 0.16 | 0.00 | 0.00 | - | 0.00 | - |
| BUT08D | 0.22 | 0.35 | 0.13 | 0.00 | 0.00 | - | 0.00 | - |
| BUT09S | 0.15 | 0.27 | 0.02 | 0.00 | - | 0.00 | 0.00 | - |
| BUT09D | 0.10 | 0.30 | 0.08 | 0.03 | - | 0.13 | 0.00 | - |
| TAB01 | 0.36 | 0.63 | 0.22 | 0.20 | 0.22 | 0.63 | 0.09 | - |
| TAM01 | 0.59 | 0.49 | 0.24 | 0.16 | 0.35 | 0.69 | 0.31 | - |
| TRW02S | 0.06 | 0.13 | 0.42 | 0.00 | - | 0.00 | 0.00 | 0.00 |
| TRW02D | 0.04 | 0.00 | 0.36 | 0.00 | - | 0.00 | 0.00 | 0.00 |
| TRWN07S | 0.08 | 0.37 | 0.06 | 0.00 | 0.00 | 0.67 | 0.00 | - |
| TRWN07D | 0.05 | 0.37 | 0.07 | 0.00 | - | 0.35 | 0.01 | - |
| TRW08S | 0.04 | 0.00 | - | 0.14 | - | 0.11 | 0.00 | 0.03 |
| TRW10S | 0.05 | 0.00 | - | 0.00 | 0.00 | 0.22 | - | 0.01 |
| TRW10D | 0.01 | 0.00 | 0.00 | - | - | 0.25 | 0.00 | 0.00 |

**Table S5: Benthic cover, nine sites across three atolls with observations in 2010 and 2012**

| Category | 2010 surveys | | | 2012 surveys | | | p-value^*^ |
| --- | --- | --- | --- | --- | --- | --- | --- |
|  | Abaiang | Butaritari | Tarawa | Abaiang | Butaritari | Tarawa |  |
| Coralline Algae | 8% | 5% | 4% | 7% | 2% | 2% | 0.189 (t) |
| Dead coral / turf | 36% | 21% | 32% | 23% | 23% | 35% | 0.670 (t) |
| *Halimeda* | 16% | 3% | 2% | 40% | 13% | 2% | 0.061 (p) |
| Hard Coral | 25% | 43% | 45% | 22% | 39% | 39% | 0.525 (t) |
| Macroalgae | 0% | 0% | 0% | 0% | 0% | 0% | 0.324 (p) |
| Other Live | 0% | 0% | 8% | 0% | 0% | 12% | 0.642 (p) |
| Rubble | 11% | 10% | 6% | 4% | 8% | 4% | 0.093 (p) |
| Recent dead coral | 1% | 8% | 1% | 0% | 0% | 0% | 0.000 (p) |
| Sand | 2% | 8% | 2% | 3% | 13% | 6% | 0.266 (t) |
| *Acropora* | 1% | 48% | 1% | 1% | 51% | 2% | 0.912 (p) |
| *Astreopora* | 0% | 1% | 0% | 0% | 1% | 0% | 0.929 (p) |
| *Echinopora* | 0% | 0% | 0% | 0% | 0% | 0% | 0.876 (p) |
| *Favia* | 7% | 1% | 2% | 6% | 2% | 1% | 0.811 (p) |
| *Favites* | 0% | 0% | 0% | 1% | 0% | 0% | 0.047 (p) |
| *Fungiid* (all) | 3% | 2% | 1% | 4% | 2% | 3% | 0.405 (t) |
| *Goniastrea* | 1% | 2% | 0% | 0% | 0% | 0% | 0.047 (p) |
| *Heliopora* | 31% | 3% | 10% | 33% | 3% | 16% | 0.837 (p) |
| *Hydnophora* | 0% | 0% | 1% | 1% | 1% | 0% | 0.301 (t) |
| *Lobophyllia* | 0% | 0% | 0% | 1% | 1% | 1% | 0.021 (p) |
| *Merulina* | 1% | 0% | 0% | 0% | 0% | 0% | 0.579 (p) |
| *Montipora* | 5% | 12% | 1% | 6% | 8% | 0% | 0.592 (p) |
| *Montastrea* | 0% | 0% | 0% | 1% | 0% | 0% | 0.490 (p) |
| *Pavona* | 2% | 0% | 0% | 1% | 6% | 0% | 0.840 (p) |
| *Platygyra* | 1% | 0% | 0% | 1% | 1% | 1% | 0.945 (p) |
| *Plerogyra* | 0% | 0% | 5% | 0% | 0% | 5% | 0.788 (p) |
| *Pocillipora* | 7% | 3% | 4% | 10% | 1% | 2% | 0.783 (p) |
| *Porites* massive | 29% | 15% | 1% | 31% | 16% | 0% | 0.894 (p) |
| *Porites rus* | 0% | 4% | 72% | 0% | 2% | 67% | 0.891 (t) |
| *Faviidae* (all) | 15% | 9% | 3% | 11% | 6% | 3% | 0.322 (t) |

* 2010 vs. 2012 cover across all atolls; (t) denotes t-test; (p) denotes permutation test (see Methods)

**Table S6: Live coral as a percent of all coral (recently dead, bleached, turf-covered**

**and CCA-encrusted) in 2012 surveys at 10-12 m depth, for identifiable taxa**

| **Category** | **Abaiang** | **Butaritari** | **Tarawa** |
| --- | --- | --- | --- |
| *Acropora* | 80.0% ± 28.9% | 64.9% ± 11.8% | 54.5% ± 15.7% |
| *Heliopora* | 73.5% ± 6.0% | 71.9% ± 27.4% | 86.7% ± 11.8% |
| *Pocillipora* | 60.5% ± 2.7% | 60.8% ± 21.3% | 43.3% ± 27.1% |
| *Porites rus* | 100.0% | 97.8% | 96.1% ± 2.2% |
| All coral | 41.4% ± 14.8% | 57.9% ± 9.8% | 57.0% ± 23.2% |


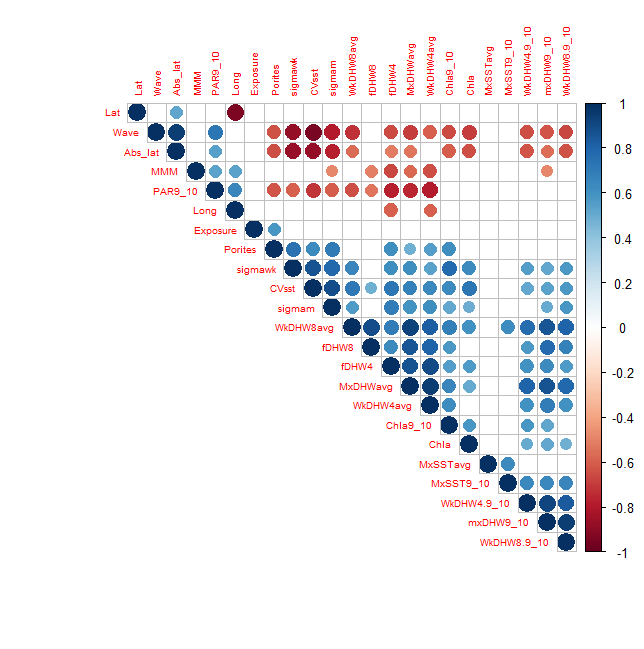
Figure S1. Correlogram of individual physical and biological variables across the 2010 field sites. Blue (red) indicates positive (negative) correlation; size of circles and shading are scaled with the correlation coefficient.
